# Rational Design Approach for Enhancing Higher-Mode Response of a Microcantilever in Vibro-Impacting Mode

**DOI:** 10.3390/s17122884

**Published:** 2017-12-12

**Authors:** Ieva Migliniene, Vytautas Ostasevicius, Rimvydas Gaidys, Rolanas Dauksevicius, Giedrius Janusas, Vytautas Jurenas, Povilas Krasauskas

**Affiliations:** 1 Institute of Mechatronics, Kaunas University of Technology, Studentu 56-123, LT-51368 Kaunas, Lithuania; ieva.milasauskaite@gmail.com (I.M.); vytautas.ostasevicius@ktu.lt (V.O.); rimvydas.gaidys@ktu.lt (R.G.); rolanas.dauksevicius@ktu.lt (R.D.); vytautas.jurenas@ktu.lt (V.J.); povilas.krasauskas@ktu.lt (P.K.); 2 Faculty of Mechanical Engineering and Design, Kaunas University of Technology, Studentu 56-338, LT-51368 Kaunas, Lithuania

**Keywords:** vibration energy harvesting, piezoelectric microcantilever, vibro-impact system, modal analysis

## Abstract

This paper proposes an approach for designing an efficient vibration energy harvester based on a vibro-impacting piezoelectric microcantilever with a geometric shape that has been rationally modified in accordance with results of dynamic optimization. The design goal is to increase the amplitudes of higher-order vibration modes induced during the vibro-impact response of the piezoelectric transducer, thereby providing a means to improve the energy conversion efficiency and power output. A rational configuration of the energy harvester is proposed and it is demonstrated that the new design retains essential modal characteristics of the optimal microcantilever structures, further providing the added benefit of less costly fabrication. The effects of structural dynamics associated with advantageous exploitation of higher vibration modes are analyzed experimentally by means of laser vibrometry as well as numerically via transient simulations of microcantilever response to random excitation. Electrical characterization results indicate that the proposed harvester outperforms its conventional counterpart (based on the microcantilever of the constant cross-section) in terms of generated electrical output. Reported results may serve for the development of impact-type micropower generators with harvesting performance that is enhanced by virtue of self-excitation of large intensity higher-order mode responses when the piezoelectric transducer is subjected to relatively low-frequency excitation with strongly variable vibration magnitudes.

## 1. Introduction

Interest in the field of vibration energy harvesting has been continuously increasing over the last decade. Many vibration energy harvesters (VEHs) are designed to power wireless sensors, thereby aiming to replace batteries which suffer from a finite lifespan and pose environmental issues. The vast majority of reported VEH designs are based on elastic structures covered with piezoelectric layers, commonly configured as uni- or bi-morphs. A literature review reveals that relatively little research has been conducted on structural optimization of microcantilever-type vibro-impacting VEHs with thorough dynamic response studies being scarce. Investigation of dynamics-related performance parameters of vibro-impact systems (VIS), such as operation speed, stability, reliability, and longevity, is a high priority topic among the other research work conducted in the field of VEHs. Designing a commercially viable device is possible only through in-depth understanding and accurate prediction of its vibrational behavior.

Dynamic characteristics of vibro-impact systems are influenced by various structural parameters. Wang and Wu [1] have employed numerical methods to determine the influence of clearance, damping, and cubic nonlinearities on the dynamic characteristics of the system. Reported research work on VIS dynamics is also concerned with the methodology to automatically choose the measurement locations of a nonlinear structure that needs to be monitored while operating [2]; deriving piecewise-linear models with a single degree of freedom for a driven vertical cantilever with a localized mass and symmetric stops [3]; introducing a modelling framework that is suitable to resolve singularities of impact phenomena encountered in practical applications [4]; and developing novel optimized microcantilevers with enhanced travel ranges [5].

Researchers working in the field of vibration energy harvesters have proposed a number of different designs and approaches to improve power generation efficiency [6,7,8,9,10,11,12,13,14]. Patel [6] developed a versatile linear model that accurately predicts the performance of cantilever-type piezoelectric VEH. Barton and Burrow [7] performed experiments with nonlinear VEHs using the control-based continuation method, which resulted in a number of different device configurations. Khovanova and Khovanov [8] conducted a comparative analysis of linear and nonlinear piezoelectric VEHs subjected to random impulsive excitations modeled by white Poisson noise. Jacquelin et al. [9] studied a piezoelectric impact-type VEH device consisting of two piezoelectric beams and a seismic mass with the final aim to optimize its performance. Liu et al. [10] analytically and experimentally investigated the wideband frequency response of a piezoelectric VEH system with stoppers on one side and two sides. Li et al. [11] adopted design approaches different from common cantilever-based configuration. They presented a practical acoustic energy harvesting mechanism for relatively low audible frequencies using the quarter-wavelength straight tube resonator with multiple PZT cantilever plates, while [12] reported VEH based on solid–solid phononic crystal and piezoelectric material. Olszewski et al. [13] created and analyzed a MEMS piezoelectric energy harvester designed to scavenge energy from the magnetic field surrounding an AC power line. Zhang [14] proposed design optimization of a single piezoelectric bimorph generator using a genetic algorithm. Vullers et al. [15] generalized energy harvesting components and their power management circuits. They have concluded that management circuits for micro-power sources have received comparatively little attention such that progress is required in order to decrease the percentage of the generated power used for power management. Roundy and Wright [16] considered the modeling, design, and optimization of a piezoelectric VEH. The proposed model of the cantilever-type device with end mass aimed to lower the 1st natural frequency. This allowed simplification of the distributed parameter system to a single degree of freedom system where higher vibration modes were not considered.

Meanwhile, current research aims to reduce the 1st natural frequency of the microcantilever-type VEH by treating its structure as a distributed parameter system that is characterized by variable cross-sections, which are distributed along the length of the cantilever using a dynamic optimization procedure. It is expected that pre-defined modes of settled transverse vibrations can be excited by employing optimal structures, which could lead to improved power generation in energy harvesting applications.

## 2. Enhanced VEH Configuration

The initial step of enhancing micro-cantilevered VEH configuration was associated with the shape optimization of VEH substrate structure. The aim of the optimization was to select such geometrical parameters that would correspond to technical characteristics of the considered dynamic system and give a minimum value to a certain quality function (a very common example would be system mass minimization with the constraint of a prescribed vibration frequency). It was also important to distinguish geometrical and structural performance constraints in order to avoid irrational structure configurations.

The target function of optimization of the VEH microcantilever substrate is expressed as:

(1) $$\Phi (A)=\min\rho \;a(A_{1}+A_{2}+\ldots +A_{m}),$$

where *A_i_* is cross-sections of structure components, *i* = 1, 2, *m*. If one employs the method of nonlinear programming (e.g., gradient projection), the following inequality-shaped constraints should be incorporated:

(2) $$\begin{array}{l} f_{m}(A)=1-A_{m}/A_{i}\leq 0,\\ f_{m+1}(\omega )=1-\omega /\omega *\leq 0 \end{array}$$

where ω* is the prescribed frequency of structural vibrations.

**Figure 1:**
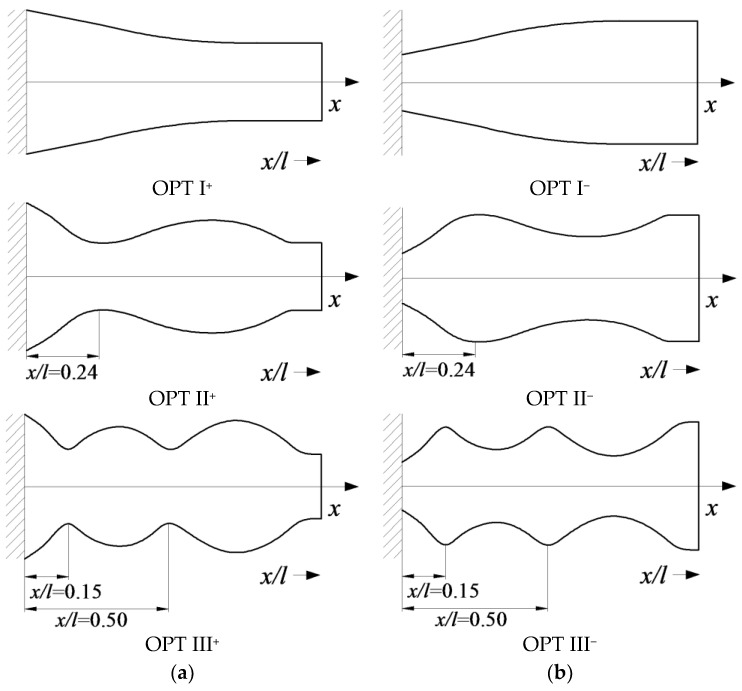
Optimal cantilever structures for the operation at increased (**a**) and reduced (**b**) natural frequencies of transverse vibrations (from top to bottom: the 1st (OPT I), the 2nd (OPT II) and the 3rd (OPT III) natural frequencies).

Optimal VEH substrate configurations obtained with a gradient projection method are presented in Figure 1. Figure 1a illustrates the optimal structures obtained for the operation at the prescribed 1st (OPT I^+^), 2nd (OPT II^+^), and 3rd (OPT III^+^) natural frequencies. These optimal microcantilever structures would attain increased natural frequencies if compared to their counterparts with constant cross-sections. Should one like to use microcantilevers with reduced natural frequencies (with respect to the constant cross-section versions), symmetrically inverted optimal structures would be obtained, as depicted in Figure 1b for the operation in the 1st (OPT I^−^), 2nd (OPT II^−^), and 3rd (OPT III^−^) natural frequencies.

Examination of the optimal VEH microcantilever substrates in Figure 1 reveals that a change of natural frequency leads to an increase in the number of cross-sectional minima/maxima along the length of the structure. Moreover, distances from the minimum and maximum cross-sections to the clamping site may be easily determined. For example, for the substrate structure that is optimal for operation at the increased 2nd frequency (OPT II^+^), the minimum cross-section is always located at the distance of 0.24 *l* from the clamping site, whereas for the structure that is optimal for operation at the increased 3rd frequency (OPT III^+^), the distance is 0.15 *l* and 0.5 *l*, respectively (*l* is the microcantilever length). A more detailed analysis of optimal microcantilever structures is presented in Figure 1, which reveals that the recurrence of maximum and minimum cross-sections corresponds to the positions of particular (maximum amplitude and nodal) points of the respective transverse vibration modes.

**Figure 2:**
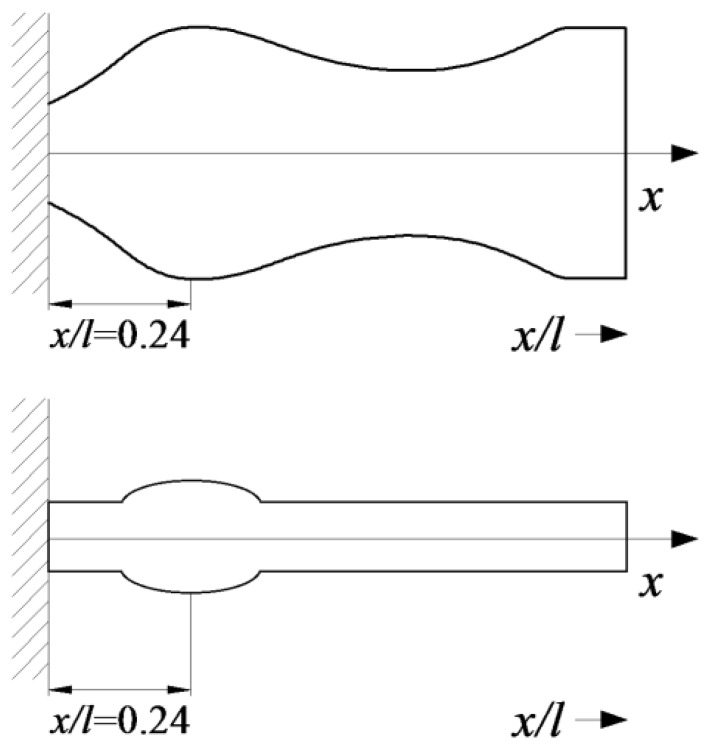
Schematics of the optimal OPT II^−^ (**top**) and the proposed rational (**bottom**) vibration energy harvester (VEH) substrate configurations.

The second step in VEH configuration enhancement is to adjust its configuration to have two separate piezoelectric layers. Harvesting performance of VEHs with continuous piezoelectric layers degrades when excited at higher natural frequencies (due to charge cancellation effects). Therefore, piezoelectric layers must be segmented at the strain nodes of higher vibration modes in order to avoid detrimental electrical cancelation effects in the piezoelectric material. Thus, a rational VEH substrate configuration was proposed (Figure 2) by taking into consideration both of the enhancement approaches described above.

The rationally-shaped substrate constitutes a structurally simplified version of the optimal microcantilever structure that is intended for operation at the reduced 2nd natural frequency (OPT II^−^). This structural simplification was performed with the objective to develop an easily manufacturable VEH configuration, which retains the prescribed dynamic characteristics of the optimal structure. Thus, the proposed rational configuration of the VEH substrate can be considered as a near-optimal version. More precisely, the intricate optimal substrate shape was replaced by a simplified design with the hump located at 0.24 *l*. Contrary to the optimal substrate structure, the rational structural configuration is amenable to conventional (micro) fabrication methods used for piezoelectric material deposition or bonding via manual assembly, and also ensures straightforward segmentation of the piezoelectric layer at the strain node of the 2nd vibration mode.

## 3. Frequency Response Measurement

### 3.1. Experimental Setup

**Figure 3:**
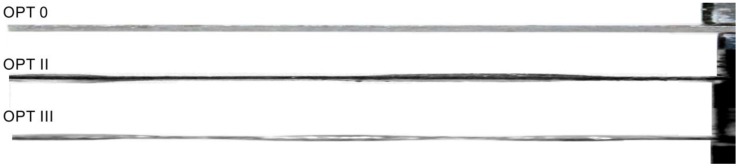
Photos of experimentally tested microcantilevers of three different configurations: OPT 0; OPT II and OPT III.

To verify the aforementioned structural VEH enhancement via dynamic testing, microcantilevers of three different configurations were fabricated from nickel using the Sigma Laser Micromachining System (Amada Miyachi America, Monrovia, CA, USA) with an accuracy of ±3 µm: the 1st configuration (OPT 0) possessed constant cross-sections; the 2nd (OPT II) and 3rd (OPT III) possessed optimal cross-sections (Figure 1b and Figure 3) for the operation at the 2nd and 3rd natural frequencies of transverse vibrations, respectively. The length of all of the cantilevers was *l* = 5 mm, width = 0.1 *l*, and the thickness varied from 0.01 *l* in the thickest part to 0.005 *l* in the thinnest part. All dimensions and measurement results are presented in dimensionless form, which allows us to compare results obtained in different scales.

**Figure 4:**
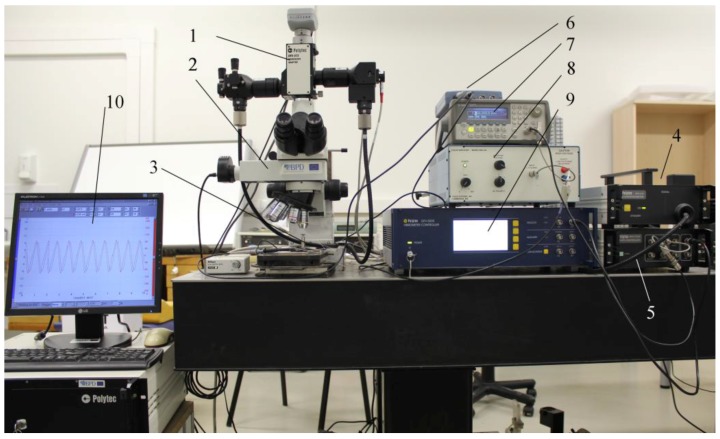
Photo of experiment setup: 1—Polytec OFV 072 Microscope adapter with Polytec OFV 073 Microscope scanner unit and Polytec OFV 071 Microscope manual positioner; 2—Nikon microscope Eclipse LV100 with digital video camera Pixelink PL-A662; 3—piezoelectric actuator PSt 150/4/20VS9 (Piezomechanik GmbH, Munich, Germany) with a researched object; 4—Polytec OFV 512 fiber-optic interferometer; 5—Polytec MSV-Z-40 Scanner controller; 6—PC oscilloscope PicoScope 3424 (PicoTechnology Ltd.,GB); 7—Function waveform generator Agilent 33220A; 8—Linear amplifier EPA-104 (Piezo Systems Inc., Woburn, MA, USA); 9—Polytec OFV 5000 Vibrometer controller; 10—Polytec Vibrascan DAQ PC.

Frequency response measurements of the fabricated cantilevers were performed using a Polytec scanning laser Doppler vibrometer (Figure 4). The tested cantilever was fixed on the platform attached to the piezoelectric actuator PSt 150/4/20VS9 (Piezomechanik GmbH, Munich, Germany), excited using a periodical signal generated by the function waveform generator Agilent 33220A, and amplified by the linear amplifier EPA-104 (Piezo Systems Inc., Woburn, MA, USA). The interference optical laser signal generated and registered by the Polytec OFV 512 fiber-optic interferometer was transformed into an electrical signal using a Polytec OFV 5000 vibrometer controller via an MSV-Z-40 Scanner controller and transmitted to a Polytec Vibrascan DAQ PC for the analysis. For the laser beam and specimen adjustment, a Nikon microscope Eclipse LV100 with the digital video camera Pixelink PL-A662 was used. The laser beam spot was positioned at the end of the cantilever. The electrical signal generated by the piezoelectric layer of the energy harvester (Section 4) was collected by an oscilloscope PicoScope 3424 (PicoTechnology Ltd., St Neots, UK).

### 3.2. Experimental Results

**Figure 5:**
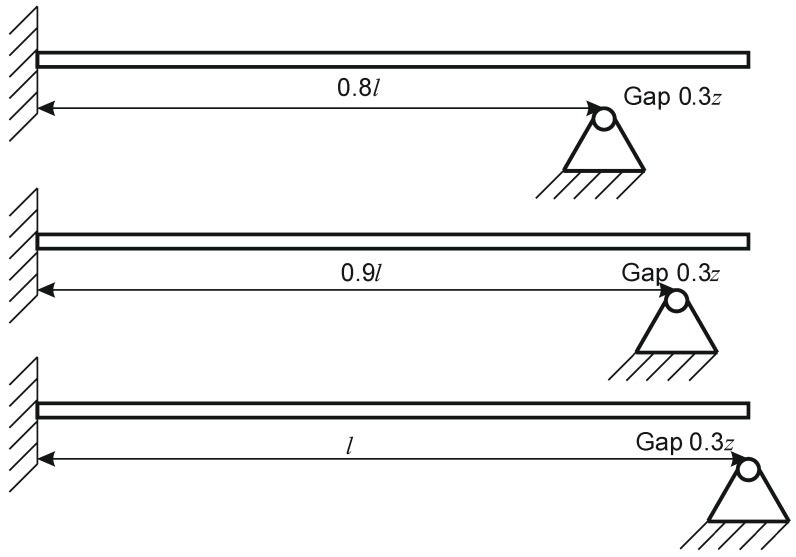
Stopper location at points approximately coinciding with the nodes of the 2nd (*x*/*l* = 0.8), 3rd (*x*/*l* = 0.9) modes of transverse vibrations and at the end of cantilever (*x*/*l* = 1).

The excitation frequency was swept in the range from 0 to 20 *ω*/*ω*_1 OPT0_ (*ω*—excitation frequency, *ω*_1 OPT0_—the 1st natural frequency of optimal microcantilever OPT 0) and amplitudes of transverse vibrations were registered, enabling the determination of the first three natural frequencies of the microcantilever. Measurements were performed with the *freely-vibrating* microcantilevers (non-impacting) as well as for vibro-impacting ones, i.e., microcantilevers impacting against a stopper located at points approximately coinciding with the nodes of the 2nd (*x*/*l* = 0.8) and 3rd (*x*/*l* = 0.9) modes of transverse vibrations of a cantilever with constant cross-sections (Figure 5).

**Figure 6:**
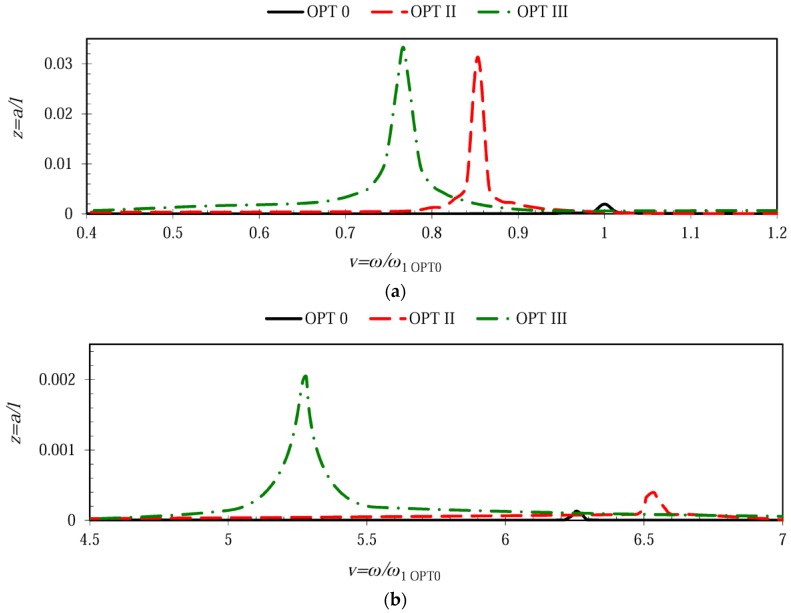
Shift of the natural frequencies for different microcantilever configurations: (**a**) shift of the 1st natural frequency; (**b**) shift of the 2nd natural frequency.

Figure 6 illustrates the shift of natural frequency for different freely-vibrating microcantilevers. The plots indicate that microcantilever OPT 0 was the stiffest one as it exhibited the highest 1st natural frequency (1.0 *ω*/*ω*_1 OPT0_) accompanied by the lowest amplitude peak. Optimal microcantilever OPT III was characterized by the lowest 1st natural frequency of 0.77 *ω*/*ω*_1 OPT0_, which constituted a 23% reduction with respect to OPT 0, while the amplitude peak was higher by more than six times. Microcantilever OPT II was somewhat in the middle between the other configurations in terms of 1st natural frequency and modal amplitude. Analysis of the shift of the 2nd natural frequency revealed that the OPT II configuration possessed a similar 2nd natural frequency (6.53 *ω*/*ω*_1 OPT0_) compared to its respective constant cross-section counterpart, OPT 0 (6.27 *ω*/*ω*_1 OPT0_). The same trend was observed for the 3rd natural frequency of microcantilever OPT III (18.24 *ω*/*ω*_1 OPT0_), which was very close to the corresponding natural frequency of OPT 0 (17.8 *ω*/*ω*_1 OPT0_).

**Figure 7:**
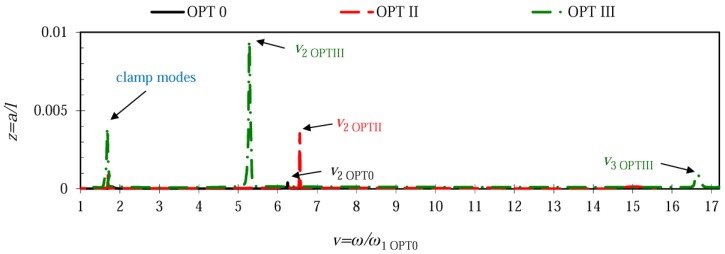
Frequency responses of different microcantilever configurations when the rigid stopper is located at *x*/*l* = 0.78 ≈ 0.8.

**Figure 8:**
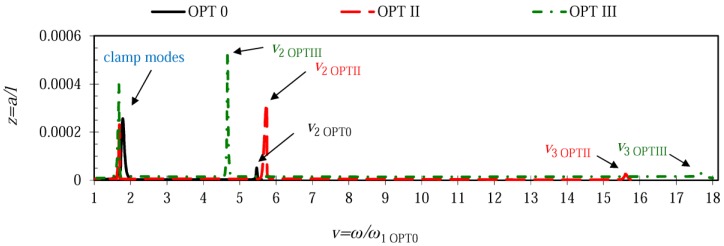
Frequency responses of different microcantilever configurations when the rigid stopper is located at *x*/*l* = 0.87 ≈ 0.9.

Figure 7 combines frequency responses of different microcantilever configurations for the case when the rigid stopper was located at *x*/*l* = 0.78 (node of the 2nd transverse mode). The plots reveal that microcantilever OPT 0 exhibited the lowest amplitudes of the 2nd mode, while OPT III was characterized by the highest vibration amplitudes for the 2nd and 3rd vibration modes with the lowest 2nd natural frequency and the highest 3rd natural frequency. Microcantilever OPT II acquired frequency and amplitude values in between other configurations. Similarly, Figure 8 provides the frequency responses of different microcantilever configurations for the case when the stopper was located at *x*/*l* = 0.87 (second node of the 3rd transverse mode). Analogously to the preceding case, microcantilever OPT III exhibited the highest vibration amplitudes of the 2nd and 3rd vibration modes, demonstrating the lowest 2nd natural frequency and the highest 3rd natural frequency. The smallest response peaks in the range of *v* from 15 to 18 indicate that both optimal microcantilever configurations OPT II and OPT III were characterized by very small amplitudes in the 3rd vibration mode.

## 4. Study of Piezoelectric Vibro-Impacting VEH Prototype Based on Rationally-Shaped Microcantilevers

Microcantilever OPT II, with an optimized cross-section for the operation at a prescribed 2nd natural frequency of transverse vibrations, was already discussed in Section 2 and Section 3. However, it is obvious that large-scale accurate fabrication of such an intricate structure with smooth variations of a cross-section would be relatively complex from the technological point of view leading to prohibitively high manufacturing costs. In addition, the straightforward covering of the curved surface of the optimal cantilever with brittle piezoceramic layers would not be possible as it requires the application of special techniques for deposition of the active layers, which would further raise the costs. Therefore, a simple-to-fabricate rational microcantilever structure (OPT RAT) was designed on the basis of optimal microcantilever OPT II^−^ (Figure 2), intended for operation at the reduced 2nd natural frequency.

**Figure 9:**
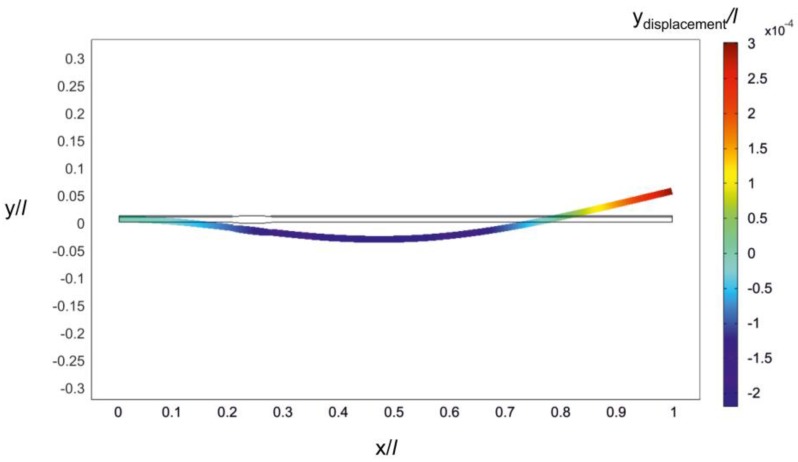
Finite element model of the proposed rationally-shaped substrate (implemented in Comsol Multiphysics).

**Figure 10:**
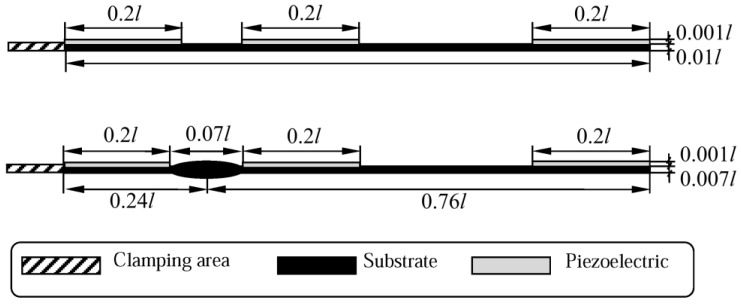
Schematics of the fabricated piezoelectric VEH prototypes: OPT 0 (**top**), rational microcantilever structure (OPT RAT) (**bottom**).

In order to determine structural parameters of the rational microcantilever, a 2D finite element model was implemented and analyzed in Comsol Multiphysics (Figure 9). The objective of the conducted simulations was to determine such geometric configuration of the OPT RAT microcantilever that would allow it to be excited at the predetermined 2nd natural frequency. Figure 10 provides structural configuration of the modeled rational microcantilever that is intended for operation in transversal (d_31_) mode. A rational design approach that is adopted here implies that an optimally-shaped zone of increased cross-section (with center located at *x*/*l* = 0.24) in the optimal microcantilever OPT II^−^ (Figure 1b) is replaced by a hump-like zone, the length of which varies from 0.01 *l* to 0.07 *l*, while sections of the structure outside the hump retain constant cross-sections (overall length and width of the microcantilever do not change with respect to the initial microcantilever of constant cross-sections).

**Figure 11:**
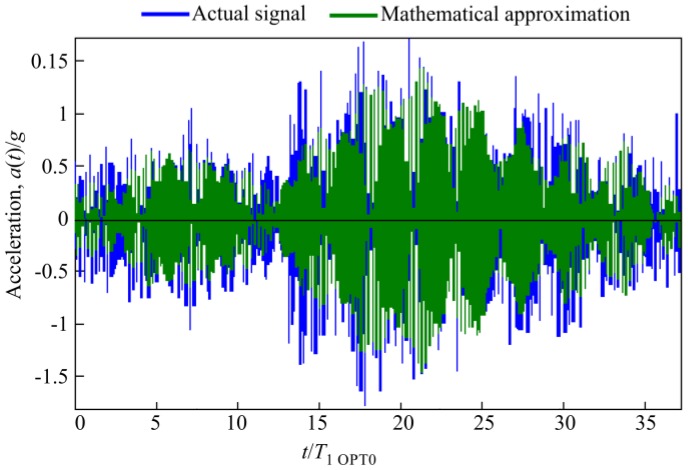
Comparison or real (**blue**) and approximated (**green**) random excitation signals (R^2^ = 0.7869).

The response of randomly excited rational microcantilever OPT RAT was determined numerically and compared with cantilever OPT 0. Both OPT RAT and OPT 0 models were subjected to random base excitations, which were defined as a vertically acting body load. In order to introduce a realistic random excitation signal to the finite element (FE) model, a transient acceleration signal (Figure 11) was measured using single-axis piezoelectric accelerometer Metra KS-93 that was mounted on an operating milling machine. A registered signal was approximated using Matlab code, which resulted in several mathematical expressions of the excitation signal, qualitatively defined by the coefficient of determination R^2^. The mathematical expression of the approximated random signal with the coefficient of determination of R^2^ = 0.7869 is presented below:

(3) $$\begin{array}{c} a(t)/g=0.01322\cdot \cos(2\pi \cdot 300t-1.6153)+0.01164\cdot \cos(2\pi \cdot 302t+0.235)+\\ 0.02796\cdot \cos(2\pi \cdot 320t-0.2856)+0.03476\cdot \cos(2\pi \cdot 322t+2.8952)+\\ 0.03383\cdot \cos(2\pi \cdot 324t-0.9567)+0.0344\cdot \cos(2\pi \cdot 3326t+1.3189)+\\ 0.00898\cdot \cos(2\pi \cdot 384t-2.2426). \end{array}.$$

An approximated random excitation signal and its comparison to the actual excitation signal are presented in Figure 11. This signal was introduced in the FE model as a vertically acting body load. Damping in FE model was evaluated using the methodology described in papers [17,18,19].

**Figure 12:**
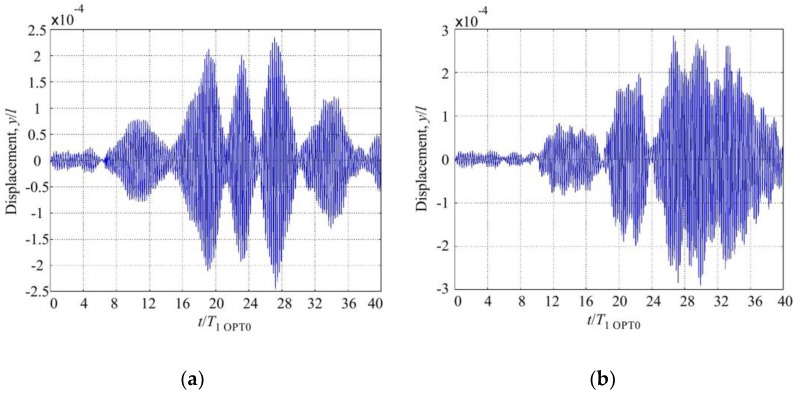
Response of the tip of randomly excited (**a**) microcantilever OPT 0 and (**b**) rational microcantilever OPT RAT.

**Figure 13:**
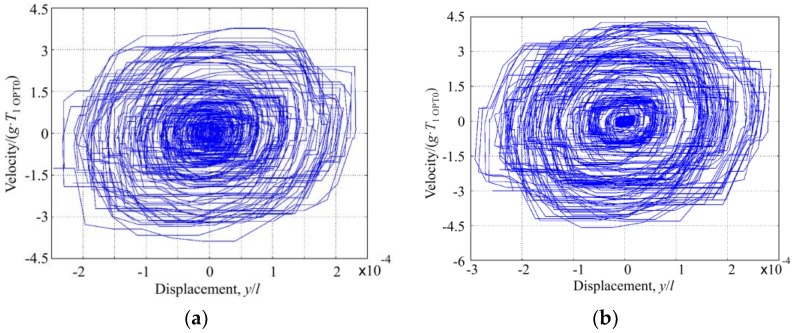
Phase diagram of the tip of randomly excited (**a**) microcantilever OPT 0 and (**b**) rational microcantilever OPT RAT.

Modeling results of the response of OPT RAT and OPT 0 microcantilevers to random excitation are presented in Figure 12 and Figure 13. The tip of the rational cantilever OPT RAT vibrates with the higher amplitude in comparison to microcantilever OPT 0. It means that microcantilever OPT RAT falls quicker into the 2nd resonant vibration shape. In addition, the vibration amplitude of microcantilever OPT RAT remains constant for the time interval that is five times longer with respect to OPT 0 case.

A stability comparison of these two microcantilevers is very clearly represented using phase diagrams (Figure 13). Curves of the phase diagram of microcantilever OPT 0 are mainly concentrated in the center, which means lower displacement and velocity as well as a lower generated mechanical energy that could be converted into electrical energy.

In addition, simulations were performed with the freely-vibrating and vibro-impacting cantilevers (stopper located at *x*/*l* = 1). In the case of the freely-vibrating cantilever, the results of a numerical modal analysis indicated that natural frequencies of OPT RAT and OPT 0 models are as follows: 1st—0.77 *ω*/*ω*_1 OPT0_, 2nd—4.52 *ω*/*ω*_1 OPT0_, 3rd—13.17 *ω*/*ω*_1 OPT0_ and 1st—1.16 *ω*/*ω*_1 OPT0_, 2nd—7.15 *ω*/*ω*_1 OPT0_, 3rd—19.9 *ω*/*ω*_1 OPT0_, respectively.

These microcantilevers were also tested experimentally. Geometric parameters of the OPT RAT model (*l* = 1 mm, width 0.1 mm) were used to fabricate a prototype of the piezoelectric VEH by employing the proposed rationally-shaped substrate made of nickel that was covered with three segments of a screen-printed piezocomposite layer (Polyvinyl butyral 20% mixed with PZT nanopowder) with electrodes [20].

**Figure 14:**
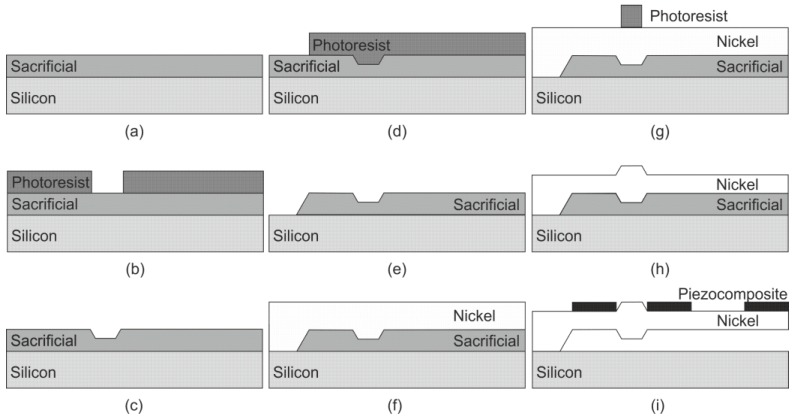
Microfabrication of microcantilever OPT RAT: (**a**) deposition of sacrificial layer; (**b**) deposition and patterning of photoresist; (**c**) patterning of sacrificial layer and removal of photoresist; (**d**) deposition and patterning of photoresist; (**e**) patterning of sacrificial layer and removal of photoresist; (**f**) deposition of nickel; (**g**) deposition and patterning of photoresist; (**h**) patterning of nickel and removal of photoresist; (**i**) screen-printing of piezocomposite and removal of sacrificial layer.

Microfabrication technology, used for the realization of microcantilever OPT RAT, is presented in Figure 14. For cantilever formation, a sacrificial layer (oxide) was deposited onto a silicon wafer using the plasma enhanced chemical vapor deposition technique (Figure 14a). The photoresist layer for the patterning of a sacrificial layer was deposited using a spin coating technique and exposed using UV lithography (Figure 14b). The exposed region was treated and used as a mask for the formation of the underlying sacrificial layer. A V-shaped groove was formed using isotropic reactive-ion etching and a photoresist was removed (Figure 14c). The new photoresist layer for the patterning of a sacrificial layer in the region of the cantilevers’ base was deposited, exposed, and treated (Figure 14d). The sacrificial layer was etched using isotropic reactive-ion etching (Figure 14e). Subsequently, a thick nickel layer was deposited (Figure 14f) and coated by a photoresist (Figure 14g). The photoresist layer was exposed using UV lithography and treated. The top surface of the cantilever was formed using isotropic reactive-ion etching (Figure 14h). Lastly, three segments of piezocomposite were screen-printed, the temporary sacrificial layer was removed (Figure 14i) and the nickel cantilever OPT RAT was free to move.

Natural frequencies of transverse vibrations of the fabricated rationally-shaped VEH were measured to be (in freely-vibrating mode) 1st—0.64 *ω*/*ω*_1 OPT0_, 2nd—3.61 *ω*/*ω*_1 OPT0_, 3rd—9.95 *ω*/*ω*_1 OPT0_. The discrepancy between the measured and simulated frequency values was attributed to non-ideal clamping of the fabricated device, which contributed to the decrease of the measured natural frequencies. A VEH prototype OPT 0 was also fabricated following the same procedure but using the nickel microcantilever of constant cross-sections as a substrate.

**Figure 15:**
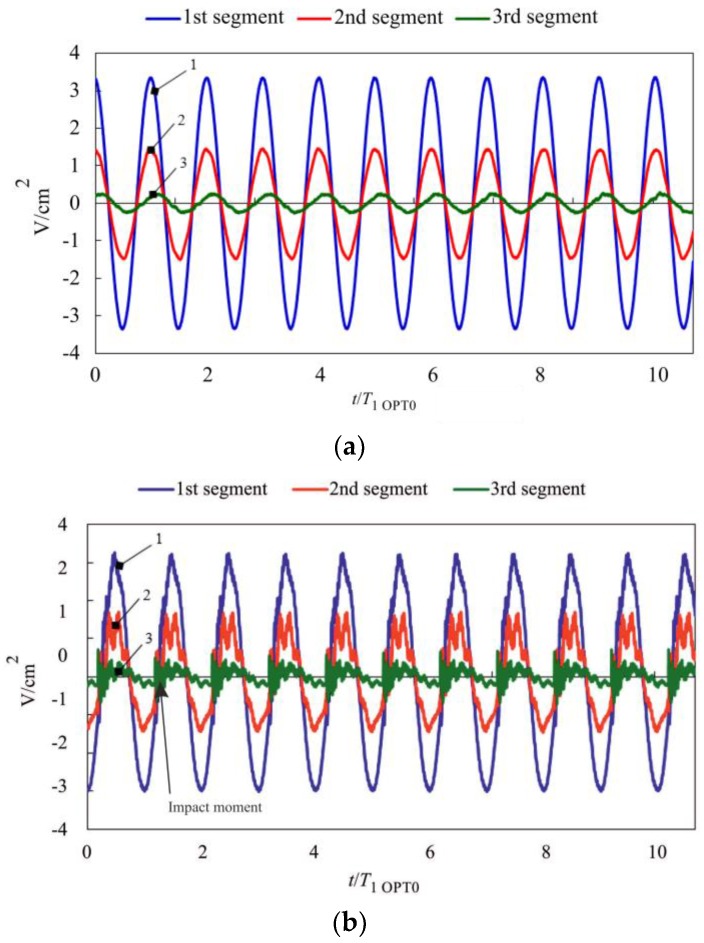
Measured open-circuit voltages for the VEH prototype OPT 0 operating in (**a**) freely vibrating mode; (**b**) vibro-impacting mode with a stopper at *x*/*l =* 1 (1st piezocomposite segment (near the clamped part of the microcantilever)—blue line (1), 2nd segment (in the middle)—red line (2), 3rd (at the free end)—green line (3)).

**Figure 16:**
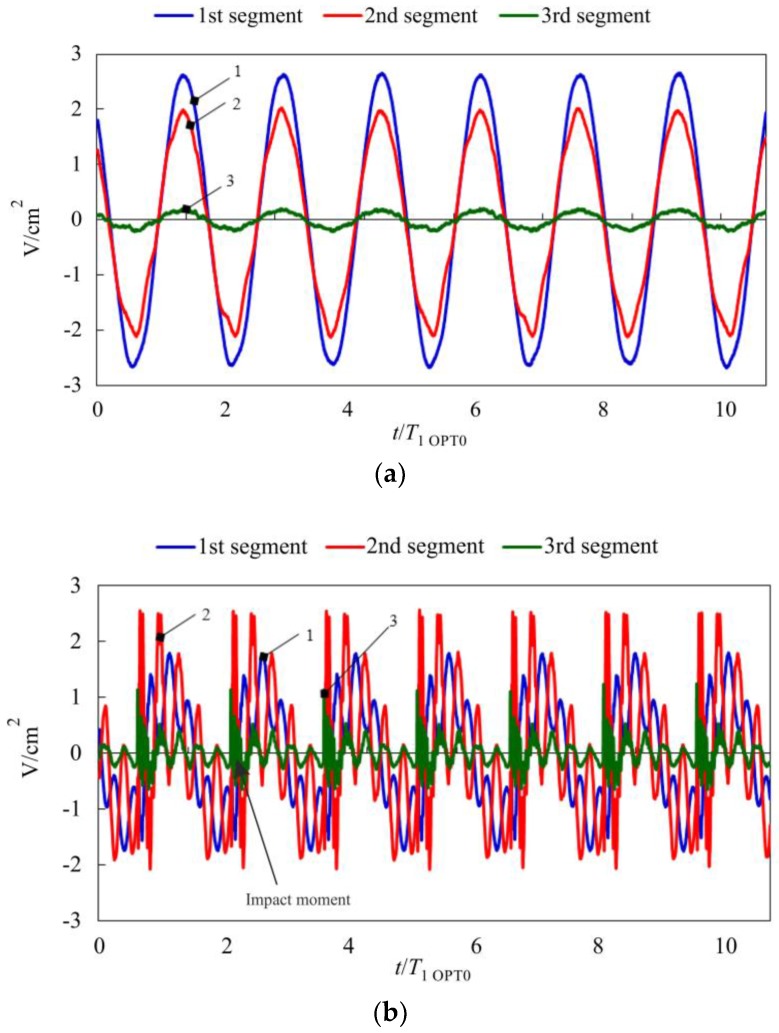
Measured open-circuit voltages for the VEH prototype OPT RAT operating in (**a**) freely vibrating mode; (**b**) vibro-impacting mode with a stopper at *x/l =* 1 (1st piezocomposite segment (near the clamped part of the microcantilever)—blue line (1), 2nd segment (in the middle)—red line (2), 3rd (at the free end)—green line (3)).

Both VEH prototypes were subjected to electrical characterization in order to compare their energy harvesting performance. Generated open-circuit voltages were measured for two cases: (i) freely-vibrating cantilever; (ii) vibro-impacting cantilever with a stopper located at the free end (*x*/*l* = 1). Figure 15 presents plots of voltage signals collected by three piezocomposite segments of the OPT 0 prototype, which was subjected to harmonic excitation at its 1st natural frequency when the microcantilever of constant cross-sections was vibrating without impacts (Figure 15a) and with impacts (Figure 15b). Meanwhile, Figure 16 provides analogous plots for the case of the OPT RAT prototype (excitation signal frequency—0.64 *ω*/*ω*_1 OPT0_). A comparison of voltage responses generated by both prototypes operating in vibro-impact mode (Figure 15b and Figure 16b) indicated a markedly larger content of higher-order harmonics in the case of OPT RAT response, though its vibration amplitudes were comparable or larger with respect to the OPT0 case. This demonstrated that the OPT RAT prototype undergoes self-excitation at higher vibration modes with the dominant 2nd mode of transverse vibrations. Thus, base excitation of the proposed rationally-shaped vibro-impacting VEH at low 1st natural frequency (0.64 *ω*/*ω*_1 OPT0_) leads to self-excitation of the vigorous vibrations at much higher frequencies (>3.6 *ω*/*ω*_1 OPT0_), which translates into higher deflection velocities and strain rates. As a result, the observed amplification of the higher-order mode response in the OPT RAT device would lead to higher power output since it is strongly dependent on the strain rate in the piezoelectric material. In addition, it would also improve the efficiency of mechanical-to-electrical energy conversion, which can be explained by means of the following relationships [21,22]:

(4) $$\eta =\frac{\zeta_{e}}{\zeta_{T}}=\frac{\zeta_{e}}{\zeta_{m}+\zeta_{e}}$$

(5) ζe=(CpωnRL)ke2(CpωnRL(ωopωn)+π2)2

where *ω_n_* and *ω_op_* are the natural and operational (excitation) frequencies, respectively; *C_p_* and $k_{e}^{2}$ are the capacitance and alternative electromechanical coupling coefficient of the piezoelectric transducer, respectively; *ζ_T_*, *ζ_m_*, *ζ_e_* are total, mechanical and electrical damping ratios, respectively.

Equations (4) and (5) indicate that an increase in vibration frequency of the piezoelectric transducer leads to stronger electrical damping (higher *ζ_e_*), which in turn improves its efficiency. This means that, with higher vibration frequencies, more mechanical energy is removed from VEH during energy harvesting process. Higher vibration frequencies of the piezoelectric transducer result in lower matched load resistance (*R_ML_* = 1/*ω_n_C_p_*), which enhances the average power generated by VEH (*P_av_* = (*V_rms_*)^2^/*R_ML_*).

## 5. Conclusions

The aim of this study was to validate an approach based on the effective exploitation of intrinsic modal characteristics of elastic structures for increasing energy harvesting efficiency of microcantilever-type piezoelectric VEH, which is configured as a vibro-impact system. The targeted applications of such nonlinear VEH include vibratory environments that are characterized by highly variable excitation levels, i.e., vibration sources for which implementation of amplitude limiters (stoppers) in a VEH is inevitable in order to retain the structural integrity of the device. The proposed rationally-shaped vibro-impacting VEH would be able to not only accommodate wide variations in excitation magnitude but would also deliver improved energy harvesting performance owing to the amplified higher-order mode responses, resulting in improved energy conversion efficiency due to increased electrical damping and higher power output due to larger response velocities of the piezoelectric transducer. The adopted design approach is referred to here as *rational* since the near-optimal shape of the piezoelectric transducer is derived on the basis of optimal cantilever structures. In other words, the rationally-designed microcantilever effectively reproduces modal behavior that is characteristic of the optimal cantilever structures (i.e., structures with a distribution of cross-sectional areas) which was determined through dynamic shape optimization. The proposed nonlinear VEH constitutes an adaptive micro-power generator, which could provide enhanced energy harvesting performance under varying real-life excitation conditions. Due to rational design modifications, the device is amenable to conventional macro-scale processing methods and advanced microfabrication methods, thereby representing a cost-effective alternative to energy harvesters that are based on complex-shaped optimal structures.
